# A Virus-like Particle-Based F4 Enterotoxigenic *Escherichia coli* Vaccine Is Inhibited by Maternally Derived Antibodies in Piglets but Generates Robust Responses in Sows

**DOI:** 10.3390/pathogens12121388

**Published:** 2023-11-24

**Authors:** Kara-Lee Aves, Priscila R. Guerra, Ana H. Fresno, Mauro M. S. Saraiva, Eric Cox, Poul J. Bækbo, Morten A. Nielsen, Adam F. Sander, John E. Olsen

**Affiliations:** 1 Department of Immunology and Microbiology, University of Copenhagen, DK-2200 Copenhagen, Denmark; 2 Department of Veterinary and Animal Sciences, University of Copenhagen, DK-1870 Frederiksberg, Denmark; 3 Laboratory of Immunology, Faculty of Veterinary Medicine, Ghent University, B-9820 Merelbeke, Belgium; 4 SEGES Innovation, Danish Pig Research Centre, Agro Food Park 15, DK-8200 Aarhus, Denmark; 5 AdaptVac, Ole Maaløes Vej 3, DK-2200 Copenhagen, Denmark

**Keywords:** F4+ enterotoxigenic *E. coli*, post-weaning diarrhea, virus-like particle, FaeG, Stb toxin, maternal antibody inhibition, sow vaccination

## Abstract

F4-positive enterotoxigenic *Escherichia coli* is associated with diarrhea and poor growth outcomes in neonatal and newly weaned piglets and is thus a major economic and welfare burden in the swine industry. Vaccination of sows with F4 fimbriae protects against the neonatal disease via passive transfer of maternal immunity. However, this strategy does not protect against infection post-weaning. Consequently, prevention and treatment methods in weaner pigs heavily rely on the use of antimicrobials. Therefore, in order to reduce antimicrobial consumption, more effective prophylactic alternatives are needed. In this study, we describe the development of a capsid virus-like particle (cVLP)-based vaccine targeting the major F4 fimbriae subunit and adhesion molecule, FaeG, and evaluate its immunogenicity in mice, piglets, and sows. cVLP-display significantly increased systemic and mucosal antibody responses towards the recombinant FaeG antigen in mice models. However, in piglets, the presence of anti-F4 maternally derived antibodies severely inhibited the induction of active humoral responses towards the FaeG antigen. This inhibition could not be overcome, even with the enhanced immunogenicity achieved via cVLP display. However, in sows, intramuscular vaccination with the FaeG.cVLP vaccine was able to generate robust IgG and IgA responses that were comparable with a commercial fimbriae-based vaccine, and which were effectively transferred to piglets via colostrum intake. These results demonstrate that cVLP display has the potential to improve the systemic humoral responses elicited against low-immunogenic antigens in pigs; however, this effect is dependent on the use of antigens, which are not the targets of pre-existing maternal immunity.

## 1. Introduction

Post-weaning diarrhea (PWD) is a common disease in industrialized pig production and presents significant economic and welfare challenges for the pig industry worldwide [1]. PWD is defined as diarrhea occurring within the first 14 days after weaning, and reports have indicated that between 10% and 90% of pigs in affected herds may experience this condition [2]. Piglets in industrialized, intensive production are typically weaned and moved to a specialized nursery unit between day 21 and day 28 after birth. At this stage, the protective immunity previously acquired from colostrum and milk intake is minimal [3]. Additionally, piglets have not yet developed a fully matured immune system, and the microbial community in their intestines is disturbed due to the rapid transition to solid feed. Collectively, these factors increase the susceptibility of newly weaned pigs to intestinal disease [4,5].

F4-positive enterotoxigenic *Escherichia coli* (ETEC) is a major contributor to PWD [6]. Colonization of the bacteria in the small intestine is mediated by the adherence of F4 fimbriae to F4-specific receptors on the brush border of swine enterocytes [7]. Following colonization, the secretion of enterotoxins, including the heat-labile (LT) and heat-stable (STa and STb) toxins, results in the loss of water and electrolytes, leading to severe diarrhea, dehydration, and reduced weight gain in the affected pig [6,8,9].

F4 infection in neonatal piglets can be prevented via vaccination of the sows with F4 fimbriae [10]. This confers colostral and lactogenic immunity to the suckling piglets and may help reduce the infectious pressure in the farrowing units. However, passive immunity does not protect against infection after weaning. Strategies to prevent and treat diarrhea in pigs post-weaning have, therefore, relied on the extensive use of medicinal zinc oxide (ZnO) and antibiotics [11,12,13]. This has led to Zn accumulation in the environment and growing concerns over the risk of antimicrobial resistance [14,15]. Consequently, increased restrictions are being imposed on the use of antimicrobials in the pig industry. As of June 2022, the use of therapeutic doses of ZnO in compound feed has been banned in Europe [16]. Without a viable alternative, it is likely that this will lead to an increase in the prevalence and severity of PWD, which, in turn, may result in an increased use of antimicrobials. 

The ETEC fimbriae are composed of polymeric assemblies of major and minor subunits, of which some are adhesion molecules [9]. In the F4 fimbriae, FaeG is the major structural subunit [7] and is the primary target of naturally acquired and current vaccine-induced immunity. FaeG is additionally responsible for adhesion to the F4 receptors, and, therefore, antibodies targeting this protein are capable of neutralizing receptor binding and preventing bacterial colonization [17,18]. FaeG has previously been investigated for use in recombinant subunit vaccines [18,19,20,21]. However, its low immunogenicity has warranted the use of multiple immunizations using high antigen doses (in the mg range), and the resulting vaccine efficacy has, nevertheless, remained inferior to that achieved using purified F4 fimbriae [18]. Additionally, the presence of anti-F4 maternally derived antibodies (MDAs) has posed a severe barrier to the effective vaccination of young piglets, even when employing the purified fimbriae approach [22]. Therefore, further investigation into methods to enhance the humoral immune response against FaeG is necessary to overcome these current challenges, which have, so far, limited effective protection against F4 ETEC, particularly during the post-weaning period.

Display of poorly immunogenic vaccine antigens on the surface of capsid virus-like particles (cVLPs) is a well-validated method for increasing antigen immunogenicity, leading to enhanced antibody responses [23,24,25]. The structural resemblance of cVLPs to native virions, including their size and particulate nature, allows effective lymphatic drainage, uptake by antigen-presenting cells, and innate immune system activation [26,27]. Furthermore, their highly ordered, repetitive surface geometry facilitates strong B cell receptor crosslinking and B cell activation [28,29]. These potent immune-stimulating properties can be harnessed for vaccine development using cVLPs as scaffolds for antigen display. The Tag/Catcher cVLP platform is a modular vaccine technology used to covalently conjugate antigens to the surface of pre-assembled AP205 bacteriophage particles [30]. Vaccine antigens are expressed and purified as a genetic fusion to a short split-protein binding Tag [31]. Upon mixing of the antigen with pre-assembled cVLPs that display the corresponding binding partner (a Catcher protein) a covalent isopeptide bond is formed between the Tag and Catcher, resulting in high-density, unidirectional antigen display. Due to its modular nature, this platform is highly versatile and has a particular advantage in being able to display complex proteins that are challenging to recombinantly produce [32], such as is the case for FaeG, which is insoluble and requires extensive purification procedures to achieve a native-like conformation [18]. 

Despite the use of unmodified cVLPs in veterinary vaccinology to target homologous viruses [33], such as in a recombinant PCV2 vaccine [34], their use as scaffolds for displaying foreign antigens has primarily been limited to treatments for chronic inflammation in companion animals [35,36,37]. Consequently, the application of cVLP display in the development of vaccines targeting infectious diseases in livestock has remained largely unexplored.

In this study, we investigated whether the display of recombinant FaeG on the surface of cVLPs could increase its immunogenicity and overcome the current limitations of F4 ETEC vaccination in piglets. We showed that cVLP display resulted in significantly higher systemic and mucosal antibody responses in mice models compared to uncoupled FaeG. However, in both suckling piglets and newly weaned pigs, the enhanced immunogenicity achieved via cVLP display was not able to overcome maternal antibody inhibition. However, at an antigen dose of less than 50 µg, the FaeG.cVLP vaccine was able to generate robust responses when used to immunize sows, inducing anti-F4 antibody responses that were on par with a commercially available vaccine.

## 2. Materials and Methods

### 2.1. Protein Expression and Purification

#### 2.1.1. FaeG.Tag Expression and Purification

FaeG, excluding the signal peptide, from F4ac *E. coli* reference strain GIS26 (GenBank: EFU2977418.1) was designed with a C-terminal 6× His-tag followed by a Gly-Gly-Ser flexible linker and SpyTag sequence (AHIVMVDAYKPTK). Flanking NcoI and NotI restriction sites were added to the gene sequence at the N- and C- terminus, respectively, and used to clone the final gene sequence into a pET-15b vector. Recombinant expression was performed in *E. coli* One Shot^®^ BL21 Star™ (DE3) cells (Invitrogen Waltham, Waltham, MA, USA) using 2× YT media with 100 µg/mL ampicillin. Media was inoculated with a 1:50 dilution of overnight (O/N) starter culture and incubated at 37 °C (180 rpm) until an OD_600_ of 0.5–0.8 was reached. Protein expression was induced by the addition of 0.1 mM isopropyl-β-D-thiogalactoside (IPTG), followed by incubation at 20 °C (180 rpm) for 16–18 h. Cells were harvested by centrifugation at 10,000× *g* for 10 min at 4 °C. The cell pellet was resuspended in lysis buffer (50 mM Tris pH 8 + 200 mM NaCl + 10% glycerol + EDTA free protease inhibitor tablets) and lyzed via sonication at 80% power for 2 × 5 min. The soluble lysate was removed via centrifugation at 42,000× *g* for 30 min. The pellet containing the insoluble protein fraction was washed twice by resuspension in 50 mM Tris pH 8 + 5% triton X-100 + 8% glucose, followed by a final wash step using 50 mM Tris pH 8 + 2 M urea, with centrifugation at 42,000× *g* for 30 min between each wash step. The final pellet was solubilized by resuspension in 50 mM Tris pH 8 + 8 M urea and incubation for 1.5 h at room temperature (RT) with agitation. Following a final centrifugation step to remove any non-solubilized material, the protein concentration was adjusted to be between 1–1.5 mg/mL with 8 M urea buffer. FaeG.Tag was refolded through the gradual removal of urea via stepwise dialysis at 4 °C. Dialysis was initiated against a buffer containing 1× PBS + 3 M urea and was subsequently diluted with 1× PBS in 6 × 25% dilution steps over 48 h. FaeG.Tag was then dialyzed against pure 1× PBS for 4 h. Precipitated proteins were removed via centrifugation, and FaeG.Tag was then further purified and up concentrated via immobilized metal affinity chromatography (IMAC) using a 5 mL HisTrap HP column (GE healthcare, Chicago, IL, USA) using 1× PBS + 60 mM or 500 mM imidazole for the binding and elution steps, respectively. Imidazole was removed via O/N dialysis against 1× PBS.

#### 2.1.2. STb.Tag Expression and Purification

The STb toxin gene (from Uniprot A0A2A3V0W5), excluding the signal sequence, was cloned with a C-terminal Gly-Gly-Ser flexible linker, followed by the SpyTag sequence, into the pETM-50 vector, immediately downstream of the DsbA gene, his-tag, and a TEV cleavage site. Recombinant expression of the DsbA-His-Tev-STb-Tag fusion protein was performed in *E. coli* One Shot^®^ BL21 Star™ (DE3) cells, as described for FaeG.Tag. After harvest, the cell pellet was resuspended in lysis buffer (50 mM Tris pH 8 + 300 mM NaCl + 0.2% Triton-X-100 + EDTA free protease inhibitor tablets) and lysed via sonication at 80% power for 2 × 5 min. The soluble lyzate was collected by centrifugation at 42,000× *g* for 30 min and then purified via IMAC on a 5 mL HisTrap HP column (GE healthcare, Chicago, IL, USA) using 50 mM Tris pH 8 + 300 mM NaCl + 60 mM or 500 mM imidazole for the binding and elution steps, respectively. Imidazole was removed via O/N dialysis against 20 mM sodium phosphate + 125 mM NaCl pH 7.4. STb.Tag was cleaved from the DsbA carrier protein by O/N incubation with TEV protease (made in house) at room temperature with gentle rotation. Precipitated proteins were removed via centrifugation, and the sample was then concentrated using a Vivaspin^®^ 20, 3.5 kDa MWCO column (Sartorius, Göttingen, Germany).

#### 2.1.3. Purification of Catcher.cVLP

The coat protein subunit of *Acinetobacter* phage AP205 genetically fused to an N-terminal SpyCatcher (Catcher.cVLP; Genbank ID: OK422508.1) was expressed and purified as previously described [30]. Briefly, Catcher.cVLP was expressed in *E. coli* One Shot^®^ BL21 (DE3) cells (Invitrogen, Waltham, MA, USA), and self-assembled particles were purified via ultracentrifugation using an Optiprep™ (Sigma-Aldrich, St Louis, MO, USA) density step gradient. Optiprep was removed via O/N dialysis against 5 L of 1× PBS using a 1000 kDa MWCO dialysis tube (Spectrum Labs, Rancho Dominguez, CA, USA). The final protein concentration was determined using a BCA assay, according to the manufacturer’s instructions (Thermo Scientific, Waltham, MA, USA).

### 2.2. cVLP Vaccine Purification and Quality Assessment

Prior to formulation, FaeG.Tag, STb.Tag, and Catcher.cVLP were LPS purified using Triton X-114 and the phase separation technique [38]. FaeG.cVLP was formulated by mixing Catcher.cVLP with FaeG.Tag at a 1:1.5 molar ratio, followed by O/N incubation at 4 °C. Excess unbound antigen was removed via ultracentrifugation using an Optiprep density step gradient followed by O/N dialysis against 1× PBS. STb.cVLP was formulated by mixing an empirically determined batch-specific ratio of STb.Tag and Catcher.cVLP and underwent O/N incubation at 4 °C. Conjugated STb.cVLP was separated from the excess unbound antigen and cleaved from its DsbA fusion partner via ultracentrifugation (as described above). To assess coupling efficiency, individual vaccine components and final vaccine formulations were run on reduced SDS–PAGE and stained with InstantBlue Coomassie staining (Abcam, Cambridge, UK). Following destaining with water, protein bands were quantified via densitometry using Image Lab software version 6.0 (Biorad, Hercules, CA, USA), and coupling efficiency was calculated by comparing the cVLP subunit band in the cVLP input lane with the same band in the FaeG.cVLP lane. Size distribution analysis was performed via dynamic light scattering (DLS). Samples were diluted to be between 0.2–0.4 mg/mL; large particles and debris were removed via centrifugation at 16,000× *g* for 2 min, and then the sample was transferred into a disposable Eppendorf UVette cuvette (Sigma-Aldrich, St Louis, MO, USA). DLS was performed using a DynaPro NanoStar (WYATT Technology, Santa Barbara, CA, USA), with measurements taken at 25 °C with 20 acquisitions of 5 s each. Average hydrodynamic diameter and percentage polydispersity (%Pd) were estimated using Dynamics software (version 7.10, WYATT Technology, Santa Barbara, CA, USA). Particle integrity was assessed using negative stain transmission electron microscopy (TEM). Samples were diluted to 0.1–0.3 mg/mL and adsorbed onto glow-discharged 200-mesh carbon-coated grids. After a 1 min incubation, the grids were washed with ultra-pure water and then stained with 2% uranyl acetate for 1 min. Excess stain was removed, and the grids were imaged using a CM 100 BioTWIN electron microscope (Phillips, Amsterdam, the Netherlands).

### 2.3. Monoclonal Antibody Binding to FaeG

Assessment of IMM01 F4 neutralizing antibody binding to recombinant FaeG.Tag was performed using an enzyme-linked immunosorbent assay (ELISA). Nunc MaxiSorp 96-well plates (Invitrogen, Waltham, MA, USA) were coated with 0.1 µg/well of recombinant FaeG.Tag, FaeG.cVLP, Catcher.cVLP, or F4ac fimbriae (purified from an *E. coli* GIS26 bacterial suspension by the University of Ghent, Ghent, Belgium) [7,18] and underwent O/N incubation at 4 °C. Plates were blocked for 1 h at RT with 0.5% skimmed milk in PBS. Thereafter, plates were incubated for 1 h with 3-fold serial dilutions of IMM01 FaeG-specific monoclonal antibody (University of Ghent, Ghent, Belgium) [18] or an isotype control, starting at a dilution of 20 ug/mL. Plates were washed 3 times with 1× PBS + 0.05% Tween 20 (PBS/T) and then incubated for 1 h at RT with goat anti-mouse IgG HRP (A16072, Life technologies, Carlsbad, CA, USA). Following 3 washes with PBS/T, plates were developed with 3,3′,5,5′-Tetramethylbenzidine (TMB) X-tra substrate (Kem-En-Tec, Taastrup, Denmark). Absorbance was measured at 450 nm using a HiPo MPP-96 microplate reader (BioSan, Riga, Latvia). The assay was performed in duplicate using two independently purified batches of FaeG.Tag.

### 2.4. Mouse Immunization Studies

#### Head-to Head Comparison of Unconjugated FaeG- vs. FaeG.cVLP-Induced Responses

Female BALB/c AnNRj mice (at 6–8 weeks old) were obtained from Janvier Labs and housed in a specific pathogen-free facility. Mice (*n* = 4–5 per group) were vaccinated intranasally (i.n) or intramuscularly (i.m) with the unconjugated FaeG.Tag or the FaeG.cVLP vaccine (in both cases, this was in the absence of an extrinsic adjuvant). Three immunizations were administered with two-week intervals. For the i.n immunization, a dose of 12 µg FaeG was used. For i.m immunizations, a dose of 5 µg FaeG was used. For i.n immunizations, mice were first anaesthetized with isoflurane, and the vaccines were administered in a volume of 30 µL using a micropipette. Serum (i.n and i.m experiments) and freshly voided fecal pellets (i.n experiments only) were collected 13 days after each immunization. 

### 2.5. Pig Immunization Studies

#### 2.5.1. Vaccination of Newly Weaned Pigs from F4-Vaccinated Sows

Newly weaned male and female piglets (4 groups with *n* = 6 per group) of approximately four weeks old (with (Landrace × Yorkshire) × Duroc genetic background) were obtained from a conventional Danish farm with SPF health status (free from *Metamycoplasma hyopneumoniae*, *Actionobacillus pleuropneumoniae*, toxigenic *Pasteurella multocida*, *Brachyspira hyodysenteriae*, PRRSV, *Sarcoptes scabiei* var. suis, and *Haematopinus suis*). Pigs were from sows that were vaccinated against ETEC (using a commercially available vaccine containing inactivated F4ac) and were themselves vaccinated against porcine circovirus 2 (PCV2; Circoflex^®^, Boehringer Ingelheim, Copenhagen, Denmark) upon arrival at the research facility. Following one week of acclimatization, the pigs (approximately 5 weeks old) were vaccinated in a prime-boost set up with a 2-week interval. The groups received either Catcher.cVLP vehicle (given with an i.m delivered prime and i.n delivered boost (i.m/i.n)), unconjugated FaeG.Tag (i.m/i.n), or FaeG.cVLP (delivered i.m/i.m or i.m/i.n in separate groups). A dose of 25 µg FaeG antigen was used. For all i.n vaccinations, a LMA Mad^®^ Nasal™ atomization device (Teleflex, Wayne, PA, USA) was employed, with an administered volume of 0.5 mL. The MAD device generates a mist of particles that are 30–100 microns in size and was verified to not negatively impact cVLP particle stability. The dosage used was based on prior experience of dosing Tag/Catcher cVLP-based vaccines in larger animals. Serum and fecal samples were collected prior to immunization and at regular intervals up to 48 days post prime immunization.

#### 2.5.2. Vaccination of Piglets from Non-F4 *E. coli*-Vaccinated Sows

Male and female suckling piglets, born from non-ETEC-vaccinated sows, were immunized i.m at approx. 2 weeks of age and boosted 11 days later on the day of weaning. Weaned pigs were moved from the production farm to the research facility and housed as described above. Piglets (*n* = 7 or 8 per group) received either PBS, FaeG.cVLP + CMS adjuvant (Litevax, Oss, the Netherlands), or Catcher.cVLP + CMS adjuvant. FaeG.cVLP was given at a maximum possible dose (based on the administered volume) of 100 µg of total protein (consisting of approx. 45 µg FaeG antigen and 30 µg Catcher protein), and Catcher.cVLP was given at a dose of 60 µg of total protein (approx. 30 µg Catcher protein). Serum samples were collected prior to immunization and 24 days post-prime immunization (2 weeks after the boost).

#### 2.5.3. Sow Vaccination

This study was carried out in a conventional Danish sow herd. The herd did not have SPF status, but was infected with mycoplasma, *Actinobacillus pleuropneumoniae* serotype 2, and PRRSV. Sows, with genetics of Landrace x Yorkshire, were inseminated with Duroc semen; hence, the piglet offspring were (LxY)xD. First litter sows (gilts) were vaccinated i.m at approximately eight and two weeks prior to farrowing. Groups (*n* = 3) received either the cVLP vaccine containing FaeG.cVLP (100 µg total protein dose) + STb.cVLP (75 µg total protein dose) + AddaVax™ adjuvant or the commercially available ETEC vaccine Porcilis^®^ ColiClos (MSD, Rahway, New Jersey, USA; given at the standard dose recommended by the manufacturer) or were left unvaccinated. Serum was collected from the sows prior to vaccination and two weeks after each immunization. Colostrum samples were collected around the time of farrowing and milk was collected two weeks later. For each litter, six large and vital piglets were selected at birth, and their serum and fecal samples were collected at farrowing and at two-week intervals up until the age of eight weeks. Piglets remained with their mother until weaning at 4 weeks old. To assess naturally acquired F4 infection, fecal swabs were taken from all piglets at weaning and from individual pigs after weaning when an incidence of diarrhea was observed.

### 2.6. Fecal Sample Preparation for Immunological Analysis

For mouse fecal samples, 3–6 freshly voided pellets were collected per mouse and stored at −80 °C until processing. Samples were weighed, and 750 µL of cold buffer (1× PBS + 1% *w*/*v* immunoglobulin free BSA + protease inhibitor tablets (Roche, Basel, Switzerland)) was added per 100 mg of feces. Samples were extensively vortexed and incubated for 4 h at 4 °C with constant agitation. Fecal extracts were then clarified via centrifugation at 10,000× *g* for 10 min at 4 °C, and the supernatant was collected and stored at −20 °C. 

For pig fecal samples, freshly voided feces were collected and stored at −80 °C until processing. Samples of between 0.5–1 mg were weighed, and 3–5 mL of cold buffer (1× PBS + 1% *w*/*v* immunoglobulin free BSA + 0.002% sodium azide + protease inhibitor tablets) was added per 1 mg of fecal material. Samples were extensively vortexed and incubated for 1 h at 4 °C with constant agitation. Fecal extracts were then clarified via centrifugation at 4000× *g* for 15 min at 4 °C, and the supernatant was collected and stored at −20 °C. 

Mouse and pig fecal extracts were normalized for total IgA using a sandwich ELISA. Ninety-six-well plates were coated with 0.1 µg/well goat anti-mouse IgA (SA5-10253, Invitrogen, Waltham, MA, USA) or goat anti-pig IgA (ab112639, Abcam, Cambridge, UK) O/N at 4 °C. Plates were blocked for 1 h at RT with 0.5% skimmed milk in PBS. Fecal extracts were added to the plates in serial dilutions from 1:500–1:5000 (mouse) and 1:250–1:2250 (pig). For mice assays, an IgA standard curve was made using a mouse IgA isotype control (14-4762-81, Invitrogen) added in 2-fold serial dilutions from 1000 ng/mL to 1 ng/mL. For pig assays, a fecal extract reference sample was added in 2-fold serial dilutions starting from a 1:250 dilution. Plates were incubated at RT for 1 h and then washed 3 times with PBS/T. Goat anti-mouse IgA HRP (62-6720, Invitrogen, Waltham, MA, USA) at a 1:1500 dilution or goat anti-pig IgA HRP (ab112746, Abcam, Cambridge, UK) at a 1:10,000 dilution was added to the plates and incubated for 1 h at RT. Plates were washed 3 times with PBS/T and developed with TMB X-tra substrate (Kem-En-Tec, Taastrup, Denmark). The absolute (mice) or relative (pig) total IgA concentration in each sample was determined from the standard curves.

### 2.7. Analysis of Vaccine-Induced Antibody Responses

An ELISA was used to determine the antigen-specific antibody responses in the serum from mice and pigs. Ninety-six-well microtiter plates (Nunc MaxiSorp, Invitrogen, Waltham, MA, USA) were coated with 0.1 µg/well of the following antigens: F4ac fimbriae (University of Ghent, Belgium) (for mouse assays), recombinant FaeG.Tag, and MBP.STb (for pig studies). Following O/N incubation at 4 °C, the plates were blocked for 1 h at RT with 0.5% skimmed milk in PBS. Serum was diluted in PBS + 0.5% skimmed milk using either a 3-fold serial dilution, starting at a 1 in 50 dilution for IgG, or a 2-fold serial dilution, starting at a 1 in 20 dilution for IgA. To evaluate seropositivity, all pig serum samples were run in parallel with a non-immune control serum sample (31890, Invitrogen). Fecal extracts were diluted to 5 µg/mL (mice) or equal relative total IgA (pigs) in PBS + 1% BSA. Sample dilutions were added to the plates and incubated for 1 h at RT. Plates were washed 3 times with PBS/T and probed for 1 h at RT with the following HRP-conjugated secondary antibodies: anti-mouse IgG (A16072, Life technologies); anti-pig IgG (ab6915, Abcam); anti-mouse IgA (62-6720, Invitrogen); anti-pig IgA (ab112746, Abcam); or unconjugated rabbit anti-swine IgM (SAB3700447, Sigma-Aldrich), followed by anti-rabbit IgG HRP (P0448, Agilent, Santa Clara, CA, USA). Plates were washed 3 times with PBS/T and developed with TMB X-tra substrate (Kem-En-Tec, Taastrup, Denmark). The absorbance was measured at 450 nm using a HiPo MPP-96 microplate reader (BioSan, Riga, Latvia). Antibody levels were reported as the area under the curve (AUC). 

### 2.8. qPCR and Culturing of Fecal Swabs

F4+ and F18+ ETEC, along with Rotavirus A, were detected in the fecal swabs via RT-qPCR, as previously described [39]. Briefly, RNA and DNA were extracted using the QIAcube HT extraction robot (QIAGEN, Hilden, Germany) and the Cador Pathogen 96 QIAcube HT kit (QIAGEN), according to the manufacturer’s instructions. The purified nucleic acid was analyzed on a BioMark HD (Fluidigm, South San Franscisco, CA, USA) and 192.24 dynamic array (DA)-integrated fluidic circuit (IFC) system (Fluidigm). To verify the RT-qPCR results, rectal swabs and diarrhea samples were streaked on blood agar plates (Oxoid, with 5% of horse blood) and incubated at 37 °C overnight. Shedding of hemolytic *E. coli* was assessed, and F4+ ETEC was confirmed via multiplex PCR analysis, as previously described [40]. 

### 2.9. Statistical Analysis

All statistical analyses were performed using GraphPad Prism version 8.4.3 (GraphPad, San Diego, San Diego, CA, USA). Differences between 2 groups were analyzed using an unadjusted, non-parametric, two-tailed Mann–Whitney U test. Differences between multiple groups were analyzed using a one-way ANOVA with a Dunnet correction for multiple testing. For all statistical tests, a *p*-value < 0.05 was deemed statistically significant.

### 2.10. Ethical Statement

All animal experiments were approved by the Danish Animal Experiments Inspectorate (Dyreforsøgstilsynet), approval numbers 2018-15-0201-01541 and 2020-15-0201-00465. All experiments were conducted in accordance with national Danish guidelines and good animal practices, as defined by FELASA.

## 3. Results

### 3.1. Development and Characterization of a cVLP-Based F4 ETEC Vaccine

**Figure 1:**
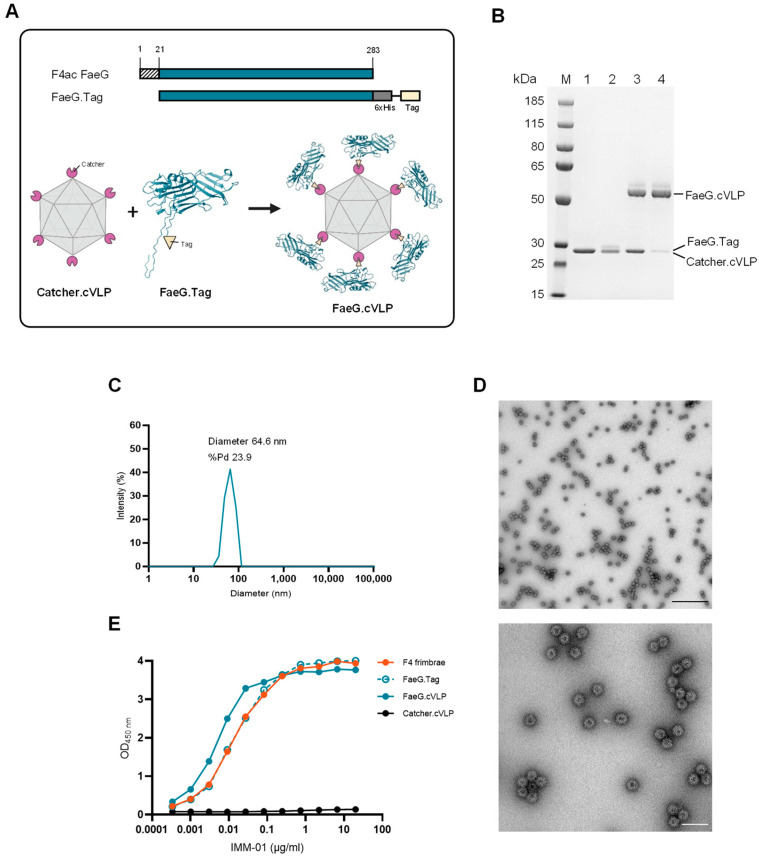
FaeG.cVLP vaccine design and characterization. (**A**) Schematic representation of FaeG antigen design and cVLP vaccine development. Full-length (FL) FaeG from F4ac *E. coli*, excluding the N-terminal secretion signal (aa1–21), was C-terminally fused to a 6× His-tag and a spilt-protein binding tag. Recombinant FaeG.Tag was mixed with Catcher.cVLPs, and the resulting covalent conjugation between the Tag and Catcher led to a unidirectional display of the antigen in the final FaeG.cVLP vaccine. (**B**) Reduced SDS–PAGE analysis of FaeG.cVLP preparation. M: molecular weight marker; lane 1: FaeG.Tag after solubilization and refolding (29 kDa); lane 2: unconjugated Catcher.cVLP subunits (27 kDa); lane 3: FaeG.cVLP following overnight incubation of FaeG.Tag and Catcher.cVLP, resulting in the formation of a coupling band (56 kDa); and lane 4: final FaeG.cVLP vaccine following the removal of excess FaeG.Tag via ultracentrifugation. (**C**) Dynamic light scattering (DLS) analysis of FaeG.cVLP. The average hydrodynamic diameter and percentage polydispersity (%Pd) is indicated. (**D**) Representative negative stain transmission electron microscopy (TEM) images of FaeG.cVLP. Scale bar = 500 nm (top); 100 nm (bottom). (**E**) ELISA data showing the binding of IMM-01 (a neutralizing anti-FaeG monoclonal antibody) to purified native F4 fimbriae, recombinant FaeG.Tag, FaeG.cVLP, and Catcher.cVLP. Results are representative of the assay run in triplicate.

To facilitate the purification and subsequent attachment of the F4 antigen to the cVLP, FaeG from the F4ac GIS26 reference strain was genetically modified to contain a C-terminal split-protein binding tag (FaeG.Tag; Figure 1A, upper panel). Following heterologous expression in *E. coli*, FaeG was purified from inclusion bodies, yielding monomers of high purity (Figure 1B, lane 1). For cVLP vaccine formulation, purified FaeG.Tag was mixed with AP205 cVLPs displaying the Catcher protein (Catcher.cVLP). The spontaneous reaction between the Tag and Catcher binding partners resulted in the formation of an isopeptide bond, enabling a unidirectional display of FaeG on the cVLP surface, forming FaeG.cVLP particles (Figure 1A, lower panel). This covalent conjugation was confirmed via SDS–PAGE analysis under reduced conditions, which showed the appearance of a protein band with a size (56 kDa) corresponding to the combined molecular weight of FaeG.Tag and the Catcher.cVLP subunit (Figure 1B, lane 3). After removal of excess FaeG.Tag antigen, the final FaeG.cVLP vaccine exhibited a high coupling efficiency (percentage of AP205 capsid subunits bound to antigen) of approximately 80–85%, indicating that the majority of the 180 available binding sites on the cVLP were occupied by the FaeG antigen (Figure 1B lane 4). Quality assessment of FaeG.cVLP was performed via dynamic light scattering (DLS) and transmission electron microscopy (TEM). A monomodal population of particles with an average hydrodynamic diameter of 64.6 nm was detected via DLS, with no indication of larger aggregates (Figure 1C). The polydispersity was, however, greater than 20%, indicating that some inter-particle interactions may have been occurring. Particle size and integrity were further confirmed via TEM (Figure 1D). 

To assess the conformation of the refolded FaeG.Tag antigen, its binding to a FaeG-specific monoclonal antibody (IMM01) was assessed via ELISA before and after conjugation to the cVLP. IMM01 binds to a conformational epitope; thus, it does not bind to denatured FaeG and has a greatly reduced affinity for misfolded FaeG [18]. Furthermore, IMM01 binds to a key epitope involved in the adhesion of F4 ETEC to F4 receptor-positive swine villi and is thereby able to inhibit this interaction [7,18]. The binding kinetics of IMM01 to FaeG.Tag was similar to that of the native FaeG from purified F4 fimbriae, and even showed enhanced binding to FaeG.cVLP (Figure 1E). No binding to Catcher.cVLP was seen, nor was a signal detected when an isotype control antibody was used instead (Figure S1). This indicates that the recombinant FaeG.Tag antigen had a native conformation around the IMM01 epitope, and that this binding site remained accessible when the antigen was displayed on the cVLP.

### 3.2. cVLP Display Increases the Immunogenicity of Recombinant FaeG

**Figure 2:**
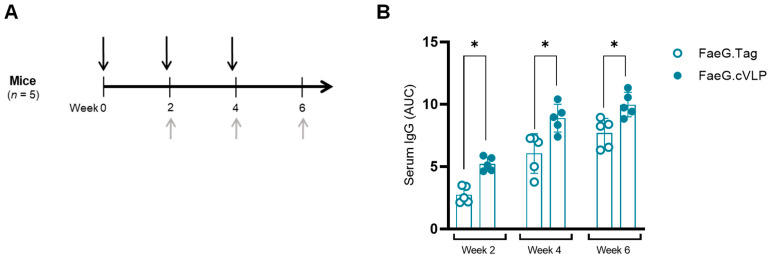
cVLP display increases the immunogenicity of FaeG in mice. (**A**) Experimental setup. Mice were immunized intramuscularly with unconjugated FaeG.Tag or FaeG.cVLP in a prime-boost-boost regime with two-week intervals (black arrows). Serum was collected 2 weeks after each vaccination (gray arrows). (**B**) ELISA results showing the anti-F4 fimbriae serum IgG response at each time point. Results show the mean area under the curve (AUC) titer ± SD at each time point. * *p* < 0.05.

The immunogenicity of FaeG.cVLP was first evaluated in mice. Mice were immunized in a prime-boost-boost regimen with either unconjugated FaeG.Tag or FaeG.cVLP, and samples was collected 2 weeks after each vaccination (Figure 2A). At each time point, when vaccinated either intramuscularly (i.m) (Figure 2B) or intranasally (i.n) (Figure S2B), FaeG.cVLP induced significantly greater levels of F4 fimbriae-specific serum IgG compared to soluble FaeG.Tag (*p* < 0.05). Similarly, when vaccinated i.n, FaeG.cVLP induced significantly greater serum and fecal IgA titers (Figure S2C,D). Thus, cVLP display was able to increase the immunogenicity of FaeG, inducing greater systemic and mucosal antibody responses after fewer doses.

### 3.3. Induction of FaeG-Specific Humoral Responses in Newly Weaned Pigs Is Hampered by Pre-Existing Maternal Antibodies

**Figure 3:**
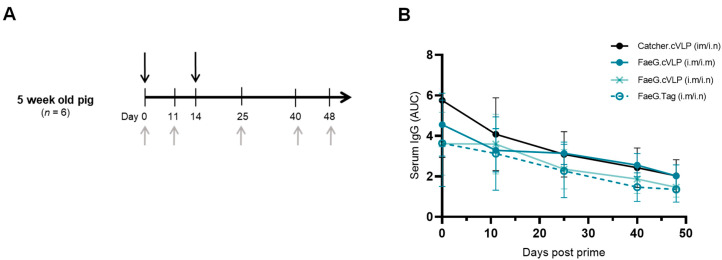
Newly weaned pigs from F4-vaccinated sows do not respond to immunization with FaeG.cVLP. (**A**) Experimental set up. Weaned pigs (*n* = 6) were immunized twice with Catcher.cVLP, FaeG.cVLP, or unconjugated FaeG.Tag using intramuscular (i.m/i.m) or heterologous intramuscular and intranasal (i.m/i.n) routes of immunization (black arrows). Serum was collected prior to the first vaccination and up to 48 days thereafter (gray arrows). (**B**) ELISA results showing the anti-FaeG-specific serum IgG response in the pigs. Results show the mean area under the curve (AUC) titer ± SD at each time point.

To assess the FaeG.cVLP-induced antibody response in pigs, weaned pigs (5 weeks old) were vaccinated with the Catcher.cVLP vaccine scaffold, unconjugated FaeG.Tag, or FaeG.cVLP in a prime-boost regimen. Serum was collected, and the anti-FaeG IgG response was followed up to 48 days post-prime (Figure 3A). The pigs were obtained from sows vaccinated with a commercial F4ac-containing ETEC vaccine. Thus, even after weaning, at the start of the study, the pigs were all seropositive and contained high FaeG-specific serum IgG titers, which were attributed to maternally derived antibodies (MDAs). These MDAs naturally declined in non-vaccinated pigs and were measurable up to the final time point taken from 12-week-old pigs (Figure S4). FaeG.cVLP vaccination of pigs with high starting MDAs did not affect the decline in FaeG-specific antibody titers, irrespective of the immunization route used (Figure 3B). In all tested groups, the average FaeG IgG titer was lower after prime-boost immunization (day 25) than at the start of the study. Furthermore, at the final tested time point (48 days post-prime), there was no statistical difference between any of the vaccinated groups and the Catcher.cVLP control group (*p* > 0.1). However, pigs vaccinated intramuscularly with the Catcher.cVLP scaffold induced robust anti-Catcher serum IgG responses that were significantly greater than that measured in pigs vaccinated with non-cVLP displayed Catcher protein (Figure S3), indicating that the cVLP scaffold itself (in the absence of conjugated FaeG antigen) was immunogenic in pigs. 

**Figure 4:**
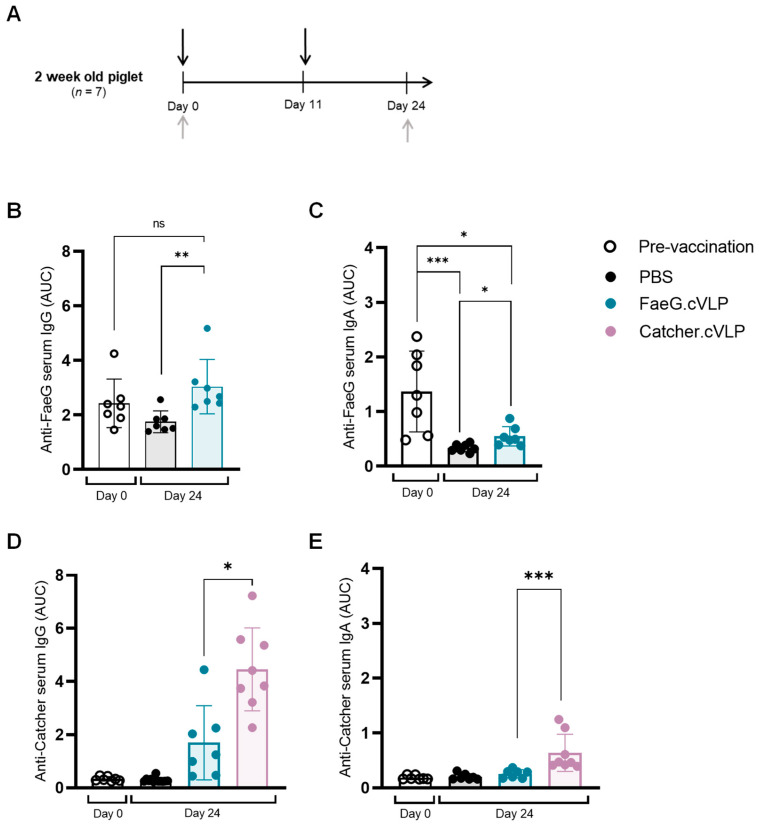
Piglets from non-F4-vaccinated sows exhibit a mild response to intramuscular immunization with FaeG.cVLP. (**A**) Experimental set up. Suckling piglets (*n* = 7) from non-F4-vaccinated sows were immunized twice intramuscularly (i.m/i.m) with PBS vehicle, FaeG.cVLP + CMS adjuvant, or Catcher.cVLP + CMS adjuvant (black arrows). Serum was collected prior to immunization (day 0) and 13 days after boost (day 24) (gray arrows). Anti-FaeG-specific IgG (**B**) and IgA (**C**), as well as anti-Catcher-specific IgG (**D**) and IgA (**E**), in the serum were measured via ELISA. Each point represents one animal; bars show the mean area under the curve (AUC) antibody titer ± SD at each time point. ns: non-significant; * *p* < 0.05; ** *p* < 0.005; and *** *p* < 0.005.

FaeG.cVLP was subsequently tested in piglets from non-F4-vaccinated sows. To enable the development of a vaccine that can induce protective responses in piglets before they become vulnerable to ETEC, it is important to assess whether an effective immune response can be induced in young piglets. Thus, in this study, 2-week-old suckling piglets were vaccinated i.m with a high dose of FaeG.cVLP (100 µg total protein) formulated with a CMS adjuvant (i.e., aiming to achieve the strongest possible immune response). An i.m boost was given on the day of weaning, and serum was collected approximately 2 weeks later (Figure 4A). Despite the sows not being actively vaccinated against F4 ETEC, all piglets were seropositive for FaeG-specific IgG at the start of the study, although the anti-FaeG antibody levels were lower than seen in piglets from conventionally vaccinated sows (Figure S4). Following prime-boost vaccination with FaeG.cVLP + CMS, there was no significant difference in anti-FaeG serum IgG compared to the titers measured at day 0 (*p* > 0.09). However, at day 24, there was a significant difference between the FaeG.cVLP-vaccinated pigs and the PBS-vaccinated controls (*p* < 0.005), indicating that the vaccine was able to compensate for the natural decline in MDAs but failed to induce a robust response (Figure 4B). Likewise, anti-FaeG serum IgA levels in the sucking piglets prior to vaccination were high and declined significantly after weaning. This drop in IgA was not prevented by FaeG.cVLP vaccination. However, at day 24, FaeG.cVLP-vaccinated piglets had a small but significantly higher serum IgA titer compared to the PBS control group (Figure 4C). These observations were also confirmed when analyzing the anti-Catcher-induced IgG response in the FaeG.cVLP + CMS-vaccinated piglets compared to the Catcher.cVLP + CMS-vaccinated piglets (receiving a comparable dose of the Catcher antigen) (Figure 4D,E). Anti-Catcher titers were present but significantly lower in the FaeG.cVLP group, indicating a general inhibition to the induction of antibodies against multiple components of the FaeG.cVLP vaccine, which is characteristic of MDA-dependent inhibition. To assess local mucosal responses, antigen-specific IgA and IgG in fecal extracts were analyzed, but were below the level of detection in all groups at both time points.

### 3.4. FaeG.cVLP Induces Robust Humoral Responses in Sows

**Figure 5:**
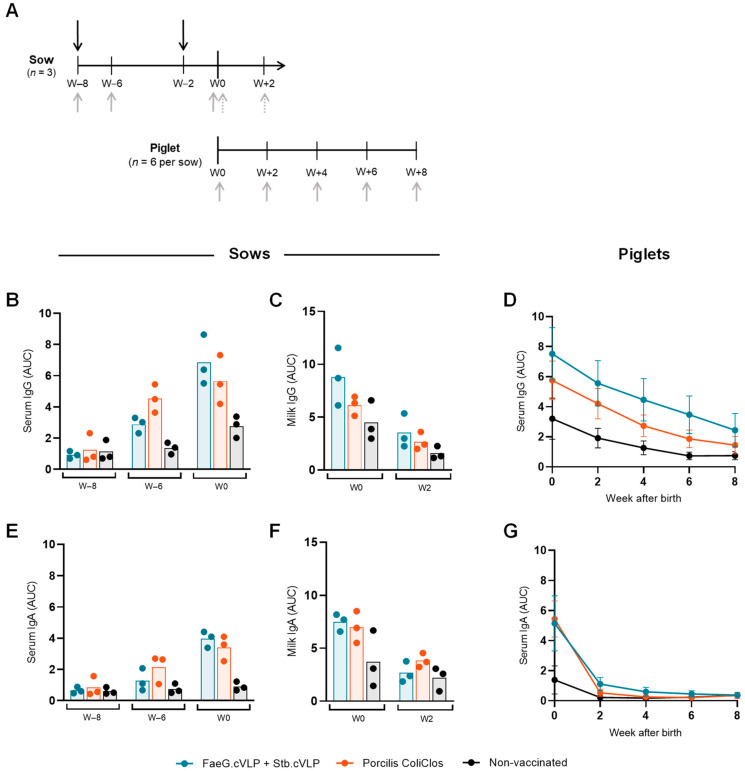
FaeG.cVLP vaccination of sows results in robust maternal antibody responses, which are passively transferred to piglets. (**A**) Experimental set up. Sows (*n* = 3) were vaccinated eight (W–8) and two (W–2) weeks prior to farrowing (W0) with either Addavax-adjuvanted FaeG.cVLP + Stb.cVLP or the commercially available Porcilis ColiClos vaccine (black arrows). A third group of non-vaccinated sows were used as controls. Serum was collected from the sows prior to immunization, two weeks post-prime, and in the week of farrowing (gray arrows). Colostrum and milk were collected from the sows at farrowing (W0) and two weeks post-farrowing (W+2), respectively (dotted gray arrows). Piglets (*n* = 6 per sow) were followed for eight weeks after farrowing. Anti-FaeG-specific IgG (**B**–**D**) and IgA (**E**–**G**) measured in the sow serum (**B**,**E**), sow colostrum/milk (**C**,**F**), and piglet serum (**D**,**G**) via ELISA. Results show the mean area under the curve (AUC) antibody titer ± SD.

As FaeG.cVLP was unable to generate robust antibody responses in piglets via active immunization, it was subsequently tested as a F4 sow vaccine, and the passive transfer of immunity to piglets via colostrum intake was measured. In this study, FaeG.cVLP was co-delivered with a second cVLP-based vaccine targeting the heat-stable ETEC enterotoxin, STb (STb.cVLP) (Figure S8). The anti-F4 antibody response was benchmarked against a conventional sow vaccine, Porcilis^®^ ColiClos, that contains, among others, the F4ac and F4ab fimbrial adhesins formulated in a dl-α-tocopheryl acetate adjuvant. Groups of three first litter sows (gilts) were vaccinated i.m with either the cVLP co-formulation or Porcilis^®^ ColiClos in a prime-boost regime at approximately 8 and 2 weeks prior to farrowing (Figure 5A). A third group of three control gilts were left unvaccinated. Serum, colostrum, and milk samples were collected from the sows and used to evaluate the antigen-specific antibody responses. Additionally, antibody levels in six piglets per sow were followed from farrowing up until eight weeks of age. 

After the prime immunization alone (week –6), FaeG.cVLP-vaccinated sows only exhibited a mild anti-FaeG response, which was lower than that induced by Porcilis^®^ ColiClos. However, at the time of farrowing (week 0), following the boost immunization, sows vaccinated with FaeG.cVLP had anti-FaeG serum IgG and IgA levels on par or marginally greater than those generated using the conventional vaccine (Figure 5B,E). These results support those shown in Figure S3, which indicate that unlike in mice, a booster immunization is required to induce robust antibody responses in pigs using the cVLP-based vaccines. In two out of the three FaeG.cVLP-vaccinated sows, the anti-FaeG IgG response in the colostrum (W0) was greater than that measured in all three conventionally vaccinated sows (Figure 5C), although the colostrum IgA level was similar between the two vaccinated groups (Figure 5F). As expected, both IgG and IgA levels in the milk samples taken two weeks after farrowing were lower in all groups than that detected in the colostrum at week 0. It should also be noted that some sows (and their piglets) in the non-vaccinated control group were seropositive for FaeG-specific antibodies, indicating that the study was run under a background of natural F4 exposure. 

Antibody titers in the piglet serum, taken shortly after farrowing (week 0), correlated well with the colostrum titers from the respective sows (Figure S5). All groups were seropositive for anti-FaeG serum IgG at week 0, which decreased with time and followed a natural parallel decay up until the final measurement, taken eight weeks after birth (Figure 5D). Similar to the trend seen in the sow colostrum (Figure 5C), two out of the three litters from FaeG.cVLP-vaccinated sows exhibited higher anti-FaeG serum IgG levels at week 0 compared to the average IgG level in the Porcilis^®^ ColiClos litters (Figure S6). In one litter in particular, robust serum IgG levels were maintained even up to 8 weeks after birth. However, due to the small number of litters used in this study, the difference in average serum IgG levels in the piglets was not significant. Serum IgA levels in the piglets were similar between the two vaccinated groups at farrowing and rapidly dropped to background levels by week 2 (Figure 5G). This rapid decay was expected, as the half-life of IgA in newborn piglets is known to be much shorter than that of IgG (estimated to only be 2–3 days) [41]. To assess the local antibody titer in the gut of the piglets, fecal samples were collected. However, at all time points, antigen-specific IgA and IgG in the fecal extracts were below the level of detection for all groups.

The anti-STb toxin antibody responses in the sows and piglets were additionally measured and followed a similar trend as seen for the anti-FaeG titers in the cVLP-vaccinated group (Figure S8). However, in general, antibody levels towards STb were lower than that detected against FaeG. 

No cases of diarrhea were observed in any of the groups prior to weaning. To assess the occurrence of natural F4+ *E. coli* infection post-weaning, piglets from all three groups were sampled on the day of weaning (Table S1), and thereafter fecal samples were collected when diarrhea was observed (Table S2). At one week post-weaning (week 5), only one F4-positive case of diarrhea was detected in the FaeG.cVLP + STb.cVLP group, while five and three cases were observed in the Porcilis^®^ ColiClos and non-vaccinated groups, respectively. However, after week 5, only two cases of F4-positive diarrhea were confirmed (one in the FaeG.cVLP + STb.cVLP group and one in the non-vaccinated group). Larger field trials will, therefore, be necessary to accurately determine the protective efficacy of FaeG.cVLP sow vaccination against F4+ *E.coli* in piglets during the neonatal and post-weaning periods.

## 4. Discussion

As regulations on the use of antimicrobials in the swine industry continue to strengthen, there is a growing need for more effective prophylactic options in order to maintain production output and animal wellbeing. F4-positive ETEC is a major contributor to diarrhea in both suckling and newly weaned piglets [42], and although sow vaccination can, in general, protect against the neonatal disease [10], effective vaccination strategies to avoid PWD are still lacking. In this study, we have, as proof-of-concept, investigated the effect of multivalent cVLP display of the main F4 antigen, FaeG, on the antibody responses induced in piglets and sows. 

We first refined a protocol for the purification of recombinant FaeG with a native-like conformation and covalently coupled it to the surface of the cVLPs using a Tag/Catcher bioconjugation approach. In mice, the cVLP display of FaeG led to a significant increase in both systemic and mucosal antibody responses compared to the unconjugated antigen alone, and higher titers were reached after fewer doses (Figure 2 and Figure S2). Likewise, in pigs, we have shown that cVLP display can enhance the immunogenicity of the model Catcher antigen when delivered intramuscularly, especially following the boost immunization (Figure S3B). However, in young pigs, the FaeG.cVLP vaccine failed to induce the superior antibody responses observed in the preliminary studies, likely due to inhibition from MDAs targeting the FaeG antigen. 

Anti-FaeG antibodies in the milk of vaccinated sows are effective in protecting neonatal piglets against F4 infection [10]. Thus, F4 ETEC vaccination of sows is a common practice in commercial farms. However, vaccine-induced IgG in the sow colostrum is transferred across the intestine shortly after birth and is present in the serum of piglets even after weaning. Following piglet vaccination, this maternally derived IgG can inhibit antigen-specific B cell activation by binding to the vaccine antigen and crosslinking the B cell receptor (which is also bound to the antigen) to the inhibitory FcγRIIB receptor (via its Fc region) (Figure S9) [43]. MDAs have been shown to inhibit antibody responses and reduce the efficacy of several human [44,45,46] and livestock [47,48,49] vaccines, irrespective of vaccine type and immunization route. Although FaeG-specific MDAs decrease in piglets with age, we were able to detect MDAs against FaeG in pigs of up to 12 weeks of age (Figure S4). Therefore, it is unlikely that an immunization schedule whereby pigs are vaccinated after MDAs have waned will be effective in providing protection prior to when the pigs are most vulnerable to F4 infection. Furthermore, anti-FaeG serum IgG was detected even in piglets from non-vaccinated sows. This is unsurprising, given that F4 ETEC is highly prevalent [6] and the FaeG antigen is an immunodominant target of naturally acquired immunity. However, it was surprising that even in the presence of these low levels of MDAs, two intramuscular vaccinations with a relatively high dose (100 µg of total protein) of FaeG.cVLP was unable to generate significant systemic antibody responses (Figure 4). Given that the multivalent nature and high epitope density of cVLPs are known to facilitate enhanced B cell receptor cross-linking, it could be hypothesized that this same structural feature of the cVLPs may also enhance the cross-linking of the BCR to FcγRIIB via the bound MDAs, potentially enhancing the inhibitory effects of the MDAs. However, this potential mechanism would need to be further validated. Additionally, it should be noted that cVLP-based vaccines are able to induce strong boosting of B cell responses in the presence of normal pre-existing immunity (i.e., boosting of an already primed response) [50]; thus, it appears that the combination of maternal IgG and the activation of naïve B cells specifically led to the inhibition observed here.

Our results are consistent with the observations of others in that F4 MDAs pose a severe barrier to the induction of actively acquired humoral immunity in piglets [51]. Despite this, the commercially available live non-pathogenic *E. coli* vaccine for piglets (ColiProtec F4/F18, Elanco) that targets F4 ETEC has shown some efficacy in increasing the performance parameters of vaccinated animals and reducing antimicrobial use [52]. However, there is no indication that this efficacy is mediated via the induction of antibodies. It is likely that the live vaccine is functioning through other mechanisms, such as outcompeting the pathogenic *E. coli* strain or lowering the opportunity for the colonization of secondary bacterial pathogens. Thus, the conclusion remains that a parenterally delivered, FaeG-containing, humoral vaccine is likely not feasible for the vaccination of seropositive piglets. Therefore, alternative PWD antigens that are not components of the current sow vaccines should be investigated. For example, in this study, FaeG.cVLP was co-formulated with a cVLP vaccine displaying the STb toxin (Figure S7). STb is one of the most prevalent virulence factors found in ETEC isolates from pigs with PWD [6]. When delivered as a soluble peptide, this toxin does not induce a detectable antibody response (Figure S7F), and thus STb greatly benefits from the cVLP display employed here. 

For the majority of this study, i.m vaccinations were employed, and serum antibody responses were measured. Although there is the possibility for some IgG to leave the blood compartment and contribute to mucosal defenses [53], in order to prevent the colonization of pathogens at the epithelial surface, secretory IgA (SIgA)-mediated mucosal immunity rather than IgG-mediated systemic responses are preferable. This requires the activation of the mucosa-associated lymphoid tissue (MALT), where antibody-secreting cells are imprinted to home back to mucosal surfaces. Administering vaccines directly to the gut mucosa via oral immunization would thus be advantageous. While oral delivery of F4 fimbriae and recombinant FaeG has been reported in weaned F4-seronegative pigs, a high dose (2–8 mg) and four consecutive immunizations were required to induce an effective response [18]. Furthermore, for most other protein-based vaccines, including cVLP-based vaccines, the harsh gastrointestinal environment leads to degradation of the ingested antigen, making oral immunization ineffective. Studies in mice [54,55,56], humans [57], and pigs [58] have shown that intranasal immunization can lead to the activation of IgA-secreting cells that home to the gut, leading to protection against enteric pathogens. Additionally, there is some evidence to suggest that i.n delivery may be a potential strategy to avoid maternal antibody interference [59,60]. In the present study, we attempted to utilize the cross-talk that exists between the different mucosal compartments by delivering the cVLP vaccines intranasally. Intranasal vaccination of mice with FaeG.cVLP led to a strong induction of antigen-specific serum IgA as well as measurable fecal IgA following the boost immunizations (Figure S2). However, i.n administration of cVLP vaccines in pigs failed to induce either a mucosal or a systemic response, even in the absence of MDAs (Figure S3B). Several factors contributing to the limited translatability of the rodent intranasal model have been previously described [61,62]. Thus, further studies will be necessary to determine why i.n immunization failed to boost responses in pigs here. This could be due to technical issues (i.e., the device, dose, or volume administered), biological factors (i.e., species-specific anatomical differences), or immunological reasons (i.e., failure of the cVLPs to fully engage with and activate the pig nasal MALT). 

As FaeG.cVLP was ineffective when used as a piglet vaccine, we subsequently evaluated its use in sow vaccination and followed the passive transfer of colostral antibodies to the suckling piglets. Following two immunizations, FaeG.cVLP induced anti-FaeG serum IgA and IgG responses in the sows that were at least on par to those induced using a commercial fimbriae-based sow vaccine (Figure 5). Unlike in mice and humans, maternal antibodies are not transferred across the placenta in pigs [63]. Therefore, the maternal immunoglobulin present in piglet serum comes almost exclusively from antibodies in the colostrum that are transferred across the intestinal barrier within the first 24 h after birth [43]. After this period, the piglet intestine becomes impermeable, and further transfer of maternal antibodies does not occur. Therefore, antibody levels in the sow colostrum serve as a primary indicator of the systemic immunity that will be transferred to the piglets. Our data clearly demonstrate this correlation. We found that serum antibody levels (both IgG and IgA) were very similar between individual piglets from the same litter, regardless of the vaccination group (Figure S5). Moreover, in two out of the three FaeG.cVLP-vaccinated sows, the anti-FaeG IgG level in the sow colostrum and the serum of their litters was notably higher than that in the three conventionally vaccinated sows and their litters. However, the group size in this study was small, which limits the extent to which precise conclusions can be drawn. Larger studies will be needed to determine whether the differences observed here are statistically significant and, importantly whether this difference can confer any additional protective benefit to the neonatal piglets. It has not been clearly established what the relative contribution of MDAs transferred via colostrum shortly after birth has on the protective efficacy of ETEC sow vaccines compared to the continuous supply of low immunoglobulin levels in the milk throughout the suckling period. However, it has been well established that the protection induced via the conventional vaccines ends almost immediately upon weaning. Therefore, it may also be worthwhile to investigate whether inducing superior colostrum responses via an alternative vaccine approach, such as the cVLP display described here, will be able to extend the time frame of passive protection into the weaning period and offer protection against both neonatal diarrhea and PWD. It should also be noted that although this study exclusively focused on anti-FaeG and STb responses, current sow ETEC vaccines (including Porcilis^®^ ColiClos) contain additional antigens which induce responses against F5 and F6 ETEC, LT toxin, and *Clostridium perfringens*. Therefore, although FaeG was used here as a proof-of-concept antigen, future candidate sow vaccines would ideally need to have a broader target range beyond F4 ETEC. 

## 5. Conclusions

In this study, we set out to investigate whether displaying the recombinant F4 adhesion protein FaeG on highly immunogenic cVLPs could improve the vaccine-induced anti-FaeG antibody responses in pigs and provide enhanced protection against PWD. Although the cVLP display significantly enhanced the immunogenicity of recombinant FaeG, effective immunization of piglets was almost completely inhibited, most likely due to the presence of maternal antibodies. However, FaeG.cVLP was able to generate strong humoral responses when utilized as a sow vaccine, and these antibodies were effectively transferred to piglets via colostrum intake. As the need to develop alternatives to the use of antimicrobials grows, vaccines targeting the bacterial causes of enteric diseases in pigs will be essential. This study provides a proof of concept for the use of cVLPs in swine vaccines. However, it also highlights several of the major challenges currently limiting piglet vaccination. While we demonstrated that the Tag/Catcher cVLP technology has the potential to enhance the systemic humoral responses elicited towards poorly immunogenic antigens in pigs, this effect is dependent on the absence of pre-existing maternal immunity. As interference by MDAs seems unavoidable for FaeG-containing vaccines, future studies should look to alternative antigen targets where pre-existing maternal immunity will not be a factor.

## Data Availability

The datasets used or analyzed in this study are available from the corresponding authors on reasonable request.

## Supplementary Materials

The following supporting information can be downloaded at: https://www.mdpi.com/article/10.3390/pathogens12121388/s1, Figure S1: Validation of the IMM-01 binding assay (related to Figure 1E); Figure S2: Intranasal FaeG.cVLP vaccination induces systemic and mucosal antibody responses in mice; Figure S3: Antibody kinetics following cVLP vaccination in mice and pigs using the Catcher protein as a model antigen; Figure S4: Level of anti-FaeG serum IgG in naïve piglets from F4 vaccinated or unvaccinated sows; Figure S5: Anti-FaeG serum antibody levels in individual litters (related to Figure 5); Figure S6: Comparison of ELISA coat proteins used to assess antibody responses; Figure S7: STb.cVLP anti-toxin vaccine; Figure S8: Anti-STb antibody responses in sows and piglets; Figure S9: Maternal antibody-dependent inhibition of FaeG.cVLP; Table S1: F4+ *E.coli* in piglets from vaccinated sows; Table S2: Incidence of diarrhea and F4-positive *E.coli* in weaned pigs from vaccinated sows.
