# Supplementary material for: A Virus-like Particle-Based F4 Enterotoxigenic Escherichia coli Vaccine Is Inhibited by Maternally Derived Antibodies in Piglets but Generates Robust Responses in Sows
Source: Pathogens. 2023 Nov 24;12(12):1388. doi: 10.3390/pathogens12121388 (PMC10745950; doi:10.3390/pathogens12121388)
Supplement: Supplementary file 1 [file pathogens-12-01388-s001.zip › pathogens-2636735-supplementary.pdf]

A virus-like particle-based F4 Enterotoxigenic *Escherichia coli* vaccine is inhibited by maternally derived antibodies in piglets but generates robust responses in sows

Supplementary Figures

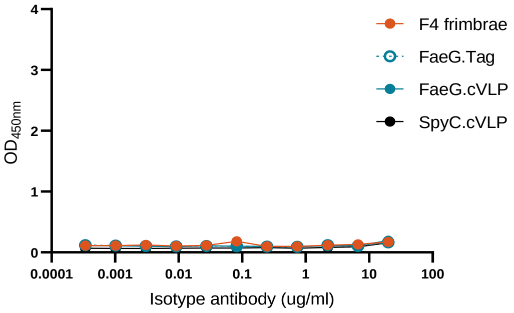

Figure S1. Validation of IMM-01 binding assay (related to Figure 1E). To demonstrate the specificity of the binding of IMM-01 neutralizing antibody to purified native F4 fimbriae, recombinant FaeG.Tag, FaeG.cVLP and the Catcher.cVLP shown in Figure 1E, the assay was repeated using an isotype control antibody. No binding was detected to any of the tested proteins.

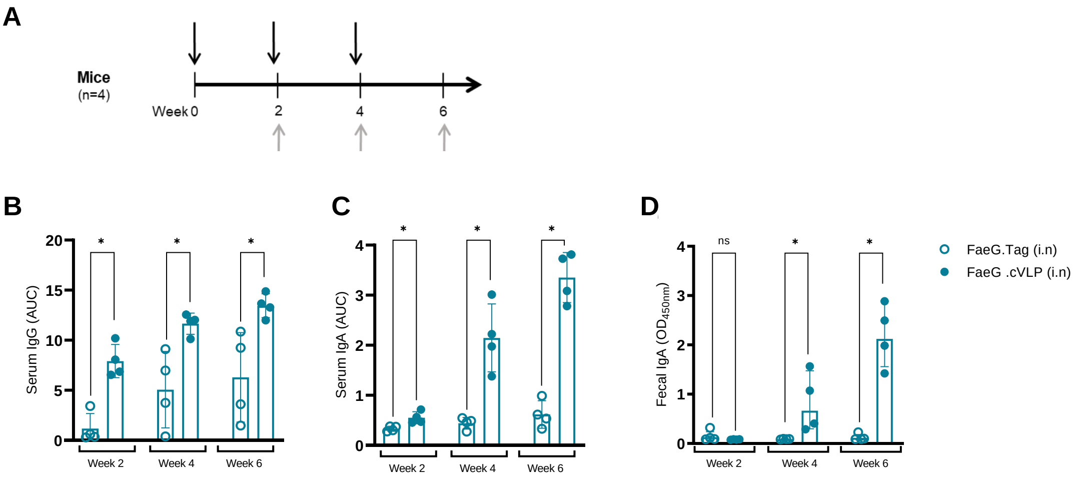

Figure S2. Intranasal FaeG.cVLP vaccination induces systemic and mucosal antibody responses in mice. (A) Experimental setup. Mice were immunized intranasally (i.n) with either unconjugated FaeG.Tag or FaeG.cVLP at two-week intervals (black arrows). Serum and fecal pellet samples were collected 2 weeks after each vaccination. F4 fimbriae specific IgG in the serum **(B)** and IgA in the serum **(C)** or fecal pellets **(D)** was measured in ELISA. Each point represents one animal, bars show the mean area under the curve (AUC) antibody titer or absorbance (OD_{450nm}) ± SD at each time point. ns: non-significant; * $p < 0.05$.

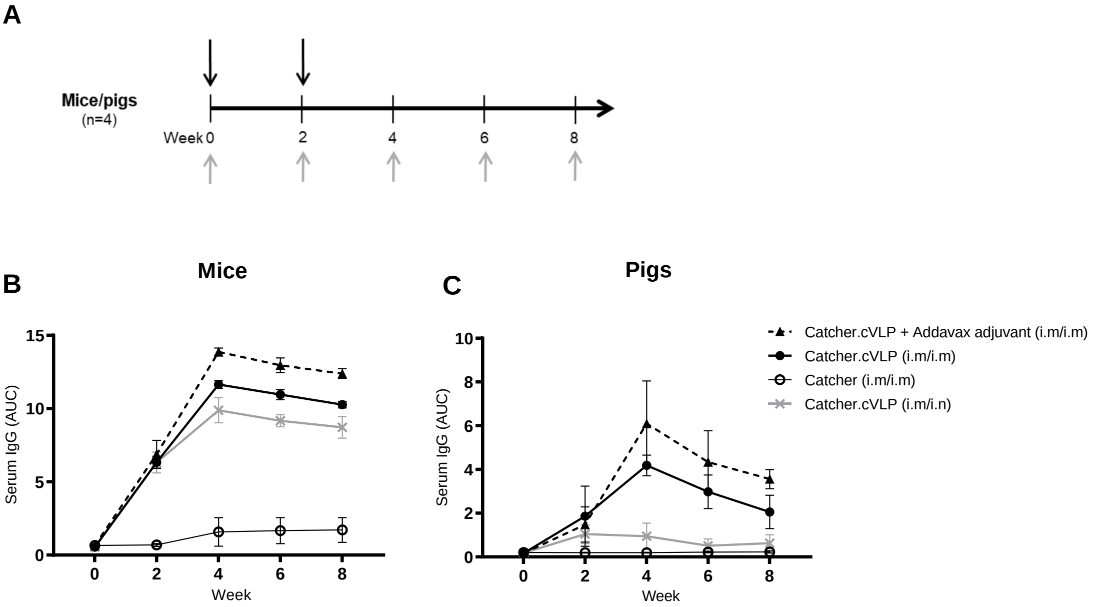

Figure S3. Antibody kinetics following cVLP vaccination in mice and pigs using the Catcher protein as a model antigen. (A) Experimental set up. Mice and five-week old weaned pigs (*n* = 4 per group) were immunized in a prime/boost set up with Catcher.cVLP or unconjugated Catcher protein at a dose of 30 µg Catcher protein (black arrows). Groups received either Catcher.cVLP formulated in Addavax adjuvant delivered i.m/i.m, Catcher.cVLP formulated without adjuvant delivered i.m/i.m or i.m/i.n and Catcher protein delivered i.m/i.m. Serum was collected prior to each immunization and at 2-week intervals for a total of 8 weeks (gray arrows). Anti-Catcher serum IgG from the mice **(B)** or pigs **(C)** was measured by ELISA. Results show the mean ± SD area under the curve (AUC) titer at each time point. As in mice, cVLP display of Catcher significantly increased the induced IgG response in pigs. However, unlike in mice, intranasal vaccination failed to boost responses in pigs. i.m: intramuscular; i.n: intranasal.

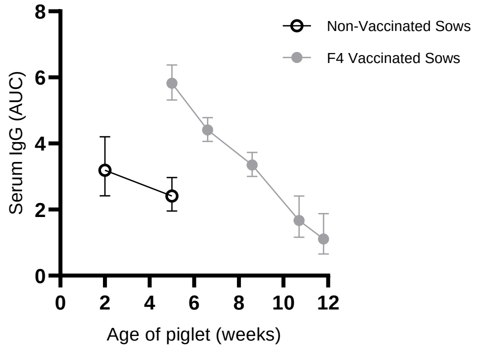

Figure S4. Level of anti-FaeG serum IgG in naïve piglets from F4 vaccinated or unvaccinated sows. Serum was collected from non-immunized piglets taken from sows that had been vaccinated for F4 *E. coli* prior to farrowing (full circles *n* = 5) or sows that had never received F4 vaccination (empty circles *n* = 6). Results show the mean ± SD area under the curve (AUC) anti-FaeG serum IgG titer.

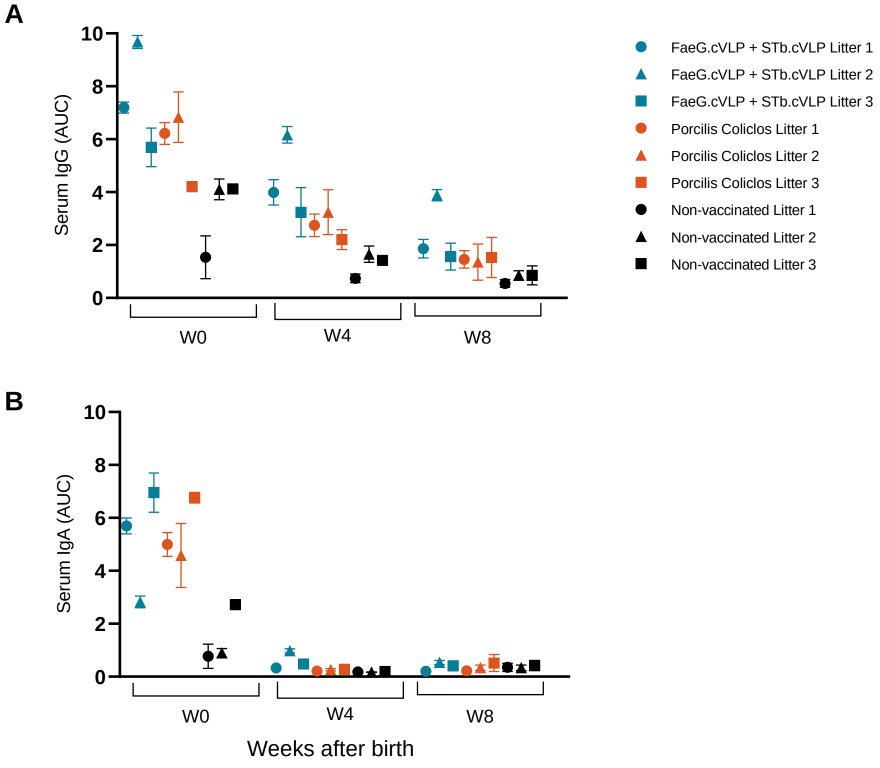

Figure S5. Anti-FaeG serum antibody levels in individual litters (related to Figure 5). Sows (*n* = 3) were vaccinated with FaeG.cVLP + STb.cVLP, Porcilis Coliclos or were not vaccinated. Their piglets (*n* = 6 per litter) were followed for 8 weeks and their anti-FaeG serum IgG **(A)** and IgA **(B)** levels were measured by ELISA. Each point represents the mean area under the curve (AUC) antibody titer ± SD from one litter.

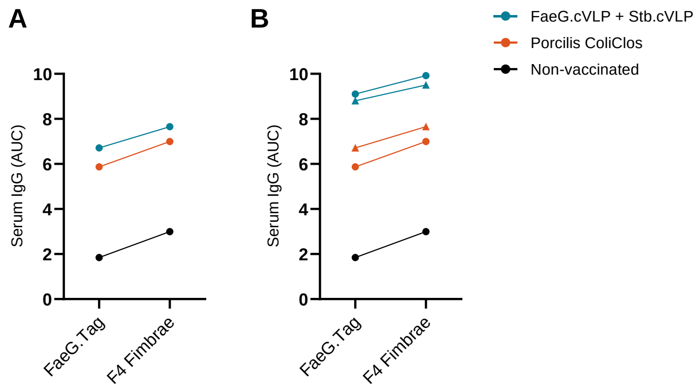

Figure S6. Comparison of ELISA coat proteins used to assess antibody responses. Selected sow **(A)** and piglet **(B)** serum samples were run in parallel in ELISA using either the recombinant FaeG.Tag or purified F4 fimbriae as the coat protein. Lines connect individual samples. The ratio of antibody AUC's between each coat is similar between the FaeG.cVLP and Porcilis Coliclos groups, indicating that there is no bias introduced by using the recombinant FaeG.Tag protein in the ELISA measurements of the sow vaccination study.

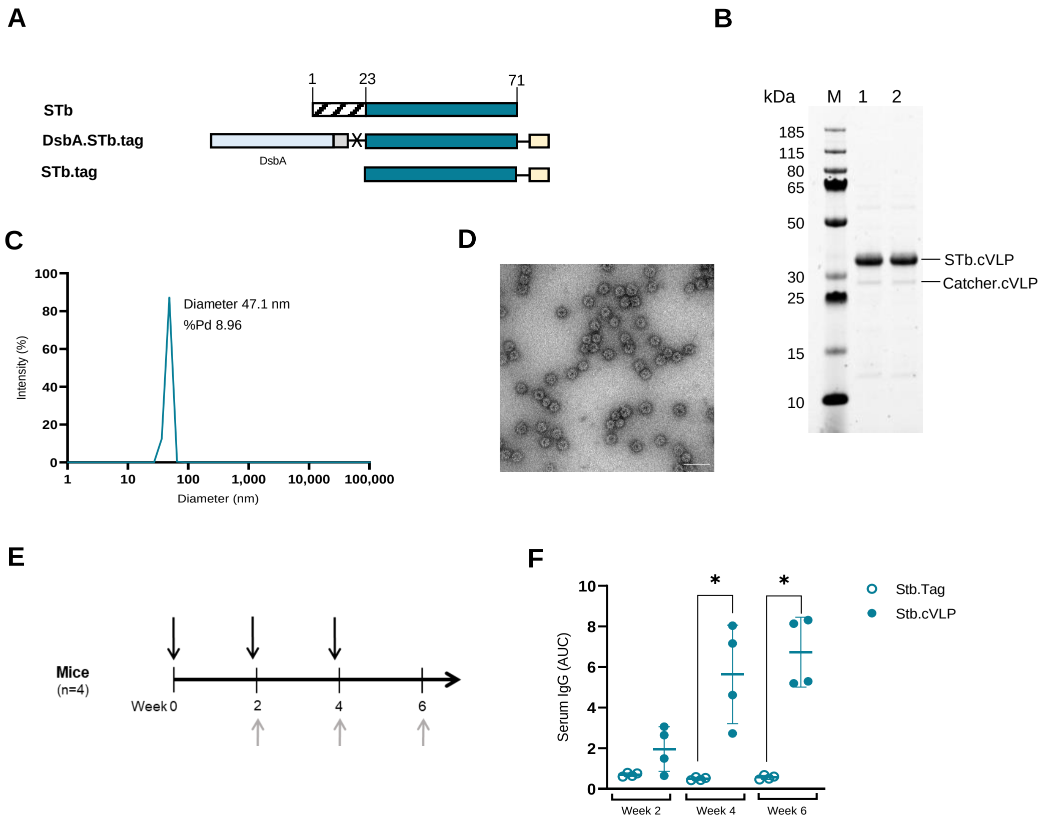

Figure S7. STb.cVLP anti-toxin vaccine. (A) Schematic representation of STb antigen design. STb excluding the N-terminal secretion signal (aa1-23), was designed with a N-terminal DsbA fusion partner (blue), 6×His-tag (gray) and C-terminal spilt-protein binding Tag (yellow). Stb-Tag was cleaved from the fusion partner via a tev cleavage site. **(B)** Reduced SDS-PAGE analysis of STb.cVLP. M: molecular weight marker, lane 1: STb.cVLP before centrifugation, lane 2: STb.cVLP after centrifugation. **(C)** Dynamic light scattering (DLS) analysis of STb.cVLP. The average hydrodynamic diameter and percentage polydispersity (%Pd) is indicated. **(D)** Representative negative stain transmission electron microscopy (TEM) images of STb.cVLP. Scale bar = 200 nm. **(E)** Experimental setup. Mice were immunized intranasally (i.n) with either unconjugated STb.Tag or STb.cVLP at two-week intervals (black arrows) at a dose of 3 µg STb antigen. Serum was collected 2 weeks after each vaccination (gray arrows). **(F)** STb specific IgG in the serum was measured in ELISA. Each point represents one animal, bars show the mean area under the curve (AUC) antibody titer ± SD at each time point. * $p < 0.05$.

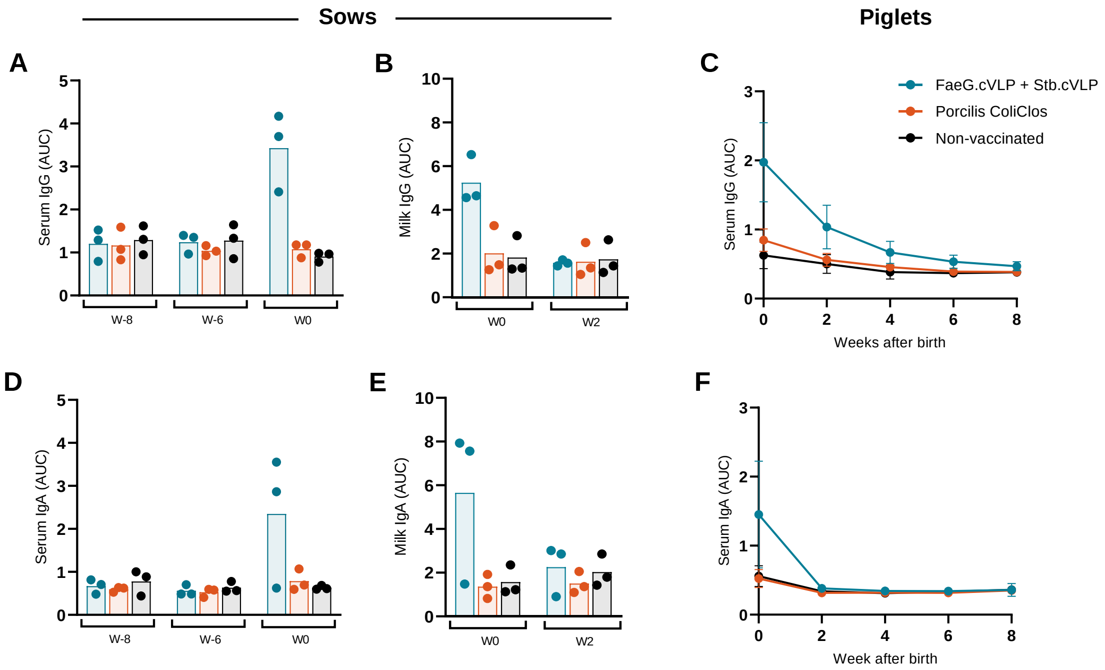

Figure S8. Anti-STb antibody responses in sows and piglets. Sows were vaccinated as described in Figure 5. Anti-STb specific IgG **(A-C)** and IgA **(D-F)** was measured in the sow serum **(A,D)**, sow milk **(B,E)** and piglet serum **(C,F)** by ELISA. Results show the mean area under the curve (AUC) antibody titer ± SD.

Table S1. F4+ *E.coli* in piglets from vaccinated sows. Piglets (n = 6 per litter) from sows vaccinated with FaeG.cVLP + STb.cVLP, Porcilis Coliclos or non-vaccinated sows were weaned at 4-weeks old. Rectal swabs were taken on the day of weaning and tested for F4+ *E.coli* using RT-qPCR. Results show the number of F4 positive piglets in each litter.

	**Week 4 (weaning)**		
	FaeG.cVLP + STb.cVLP	Porcilis Coliclos	Non-Vaccinated
Litter 1	0	0	0
Litter 2	2	0	0
Litter 3	1	1	0
**Total +ve for F4**	**3**	**1**	**0**

Table S2. Incidence of diarrhea and F4 positive *E.coli* in weaned pigs from vaccinated sows. In the four weeks following weaning, fecal samples were taken from pigs when diarrhea was observed and samples were tested for F4+ *E.coli* using RT-qPCR. Results show the number of pigs positive for F4+ *E.coli* / number of diarrhea cases in each litter.

	**Week 5**			**Week 6**			**Week 7**			**Week 8**		
	FaeG.cVLP + STb.cVLP	Porcilis Coliclos	Non-Vaccinated	FaeG.cVLP + STb.cVLP	Porcilis Coliclos	Non-Vaccinated	FaeG.cVLP + STb.cVLP	Porcilis Coliclos	Non-Vaccinated	FaeG.cVLP + STb.cVLP	Porcilis Coliclos	Non-Vaccinated
Litter 1	0 / 4	1 / 1	1 / 1	0 / 0	0 / 0	0 / 1	0 / 5*	0 / 4*	1 / 1	0 / 0	0 / 0	0 / 0
Litter 2	0 / 1	0 / 1	0 / 0	0 / 0	0 / 0	0 / 0	0 / 1	0 / 0	0 / 0	0 / 0	0 / 1	0 / 0
Litter 3	1 / 1	4 / 5	2 / 4	1 / 2	0 / 1	0 / 1	0 / 0	0 / 0	0 / 0	0 / 0	0 / 2	0 / 0
**Total +ve for F4**	**1**	**5**	**3**	**1**	**0**	**0**	**0**	**0**	**1**	**0**	**0**	**0**
**Total diarrhea cases**	**6**	**7**	**5**	**2**	**1**	**2**	**6**	**4**	**1**	**0**	**3**	**0**

* Samples were positive for Rotavirus.

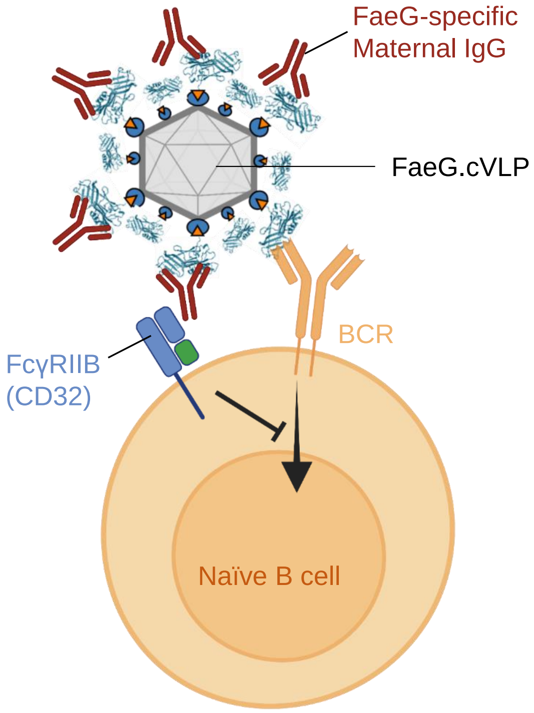

Figure S9. Maternal antibody-dependent inhibition of FaeG.cVLP. Maternally derived anti-FaeG IgG is present in commercial piglets prior to vaccination. Following intramuscular vaccination with FaeG.cVLP, this IgG binds to the FaeG antigen. Binding of FaeG to the B cell receptor (BCR) and crosslinking of BCRs would normally result in strong uptake and subsequent B cell activation. However, FcγRIIB, the inhibitory Fc receptor, is able to bind the Fc region of the maternal IgG that is coating the vaccine. This cross linking of the FcγRIIB with the BCR inhibits B cell activation and thus greatly limits the antibody responses induced by the vaccine. In the absence of MDAs, the high antigen density and multivalency achieved by cVLP display strongly promotes BCR crosslinking, thus it could be hypothesized that this antigen presentation may in fact be enhancing the FcγRIIB-BCR crosslinking in this instance compared to unconjugated antigens. Figure created with BioRender.
